# Digital Storytelling for People With Cognitive Impairment Using Available Mobile Apps: Systematic Search in App Stores and Content Analysis

**DOI:** 10.2196/64525

**Published:** 2024-10-24

**Authors:** Di Zhu, Abdullah Al Mahmud, Wei Liu, Dahua Wang

**Affiliations:** 1 Centre for Design Innovation School of Design and Architecture Swinburne University of Technology Melbourne Australia; 2 Faculty of Psychology Beijing Normal University Beijing China

**Keywords:** mobile apps, digital storytelling, older adults, mobile phone, cognitive impairment

## Abstract

**Background:**

Growing evidence suggests cognitive and social health benefits can be derived from digital storytelling for older adults with cognitive impairment. Digital storytelling apps offer the potential to serve as an on-demand, easy-to-access platform for enhancing cognitive abilities and promoting social well-being. Yet, despite the increasing quantity of such apps being available on the market, there is a gap in research investigating their quality.

**Objective:**

This app review aims to assess the digital storytelling apps available in the Chinese market and evaluate them in accordance with the Mobile Application Rating Scale (MARS). The goal was to identify key features and evaluate the overall quality in the context of cognitively impaired users.

**Methods:**

A systematic search was conducted in both the Google Play store (Google LLC) and iTunes store (Apple Inc), using English and Chinese keywords. Apps were chosen according to specific criteria that included features, including (but not limited to) memory capture, story saving, cue-based reminiscing, and the ability to share stories or memories with others. The MARS was used by 3 individual researchers to independently assess app quality across several domains, such as engagement, functionality, aesthetics, and information quality, for both Android and iOS apps.

**Results:**

From an initial screening of 297 apps, only 9 (3%) met the criteria for detailed evaluation using MARS. The reviewed apps featured *capture memory*, *save*, *reminisce*, and *share* functions, which are critical in supporting cognitive functions and enhancing user engagement. The analysis revealed patterns in platform diversity and geographical distribution of developers, with apps available on both iOS and Android. Memoirs of Life and Memorize: Diaries, Memories, Notes, Ideas, Timelines, Categories (Fair Apps Mobile) had the highest mean MARS scores of 3.35, indicating strong engagement, functionality, and information quality, while the lowest score was 2.33. The overall mean score across all apps was only 3.03 (SD 0.60), highlighting significant variation, particularly in information quality. User feedback also showed considerable variability, ranging from 0 comments for apps such as Grand Storyteller (VarIT Inc) and PWI Storyteller (Project World Impact, LLC) to as many as 5361 comments for FamilySearch, which received extensive positive reviews. This wide range of user feedback underscores the importance of continuous improvement and user-centered design, particularly in enhancing information quality and content accuracy.

**Conclusions:**

The systematic search and evaluation highlight the diverse capabilities yet variable quality of digital storytelling apps available within the Chinese market, reflecting user experiences, satisfaction levels, and efficacy in supporting cognitively impaired users. While some apps excel in engagement and functionality, others need significant improvements in information quality and user interface design to better serve those with cognitive impairments. Future research is recommended to investigate regional limitations and features that would result in more inclusive and effective digital storytelling apps.

## Introduction

### Background

Storytelling, as a practice of therapeutic intervention, has been shown to empower people with cognitive impairment engaged in chronic disease self-management, facilitating recovery by allowing them to actively identify needs and knowledge gaps while fostering connections with peers who share similar experiences [1]. For instance, a storytelling-based intervention resulted in significant improvements in blood pressure [2] among older adults diagnosed with uncontrolled hypertension. Despite the demonstrable physical benefits, storytelling apps designed for people with cognitive impairment offer a range of mental health and general well-being benefits. First, these apps serve as powerful tools for memory enhancement, with empirical studies demonstrating positive improvements in long-term and short-term memory [3,4] as well as older adults recollection and improved cognitive functions via leveraging narratives and visuals [5,6]. By engaging users in personalized and meaningful activities, including story-editing activities [7] and story-sharing activities [8], these apps contribute to a sense of purpose and accomplishment, aiding positive psychosocial and cognitive recovery. In the context of communication and social interaction, these apps play a crucial role by facilitating meaningful connections between their caregivers (including spouses) [8,9], family members [10], and peers [11,12]. Moreover, emotional connections fostered through storytelling have been found to be integral in promoting overall well-being [13], mitigating symptoms of depression and anxiety [14], and even linking older adults with dementia to personal histories, thereby aiding memory recall. These systems are crafted to evoke memories, enhance communication, and foster social engagement by leveraging digital media technologies [15,16]. There are key differences between fictional storytelling and reminiscence-based storytelling, both of which have distinct impacts on cognitive, emotional, and social processes. Fictional storytelling, for instance, has been shown to enhance cognitive engagement by transporting individuals into the narrative, thus deepening their emotional involvement and promoting cognitive stimulation [17]. By contrast, reminiscence-based storytelling plays a crucial role in memory enhancement and emotional well-being, particularly among older adults, by encouraging the recall of personal memories and fostering a sense of identity and continuity [18]. The emotional processing involved in these 2 types of storytelling also differs significantly. Fictional storytelling often leads to the creation of new emotional experiences, while reminiscence-based storytelling is more likely to evoke past emotions, reinforcing a person’s narrative identity [19]. In addition, the social benefits of reminiscence-based storytelling are well documented, with research showing that it can improve social interactions and contribute to overall well-being in older populations [15]. Research shows that individuals with cognitive impairments, including those with dementia, retain the ability to generate creative stories through structured programs. For instance, the TimeSlips program is an arts-based, creative storytelling initiative designed for people with dementia. In this program, participants collaboratively create stories using their imaginations and respond to staged pictures, guided by a facilitator [20]. This approach not only fosters creativity but also encourages meaningful social interactions among participants, highlighting their capacity to engage in generative activities despite cognitive challenges. This evidence supports the notion that even individuals with cognitive impairments can actively participate in creative storytelling, contributing to collective narratives and maintaining social connections. Thus, using the process of storytelling, these apps catalyze effective communication, supporting cognitive and psychosocial recovery, as well as enriching the lives of older adults with cognitive impairment.

Despite this potential, the plethora of storytelling apps available on the market—and their utility and efficacy for the cognitively impaired—remains woefully understudied. With a lack of concrete evidence regarding their quality, a need to fill this research gap emerges [21]. While the potential benefits for those with cognitive impairment are well understood in theory, the commercial digital storytelling apps available remain largely untested; instead, the academic focus has thus far been commercial software, primarily instant-messaging apps [22] and video editing software [7]. Given the uncertainty deriving from the plethora of available apps, consumers and older adults often turn to reviews and ratings on app stores or published on developer websites to inform their decision-making process [23]. However, reliance on such reviews brings forth the challenge of ensuring these apps are genuinely suitable for individuals with cognitive impairment. Indeed, despite the theoretical utility of such apps for older adults who are cognitively impaired, no specific measure or ranking system exists designed specifically for that user group. Extant consumer ratings were found to be poor indicators of clinical utility or usability, with a significant number of apps exhibiting potentially misleading and dangerous health information entered by users [21].

A scoping review explored studies related to digital storytelling apps published in 2023 [6], wherein only 1 study was found to have undertaken a randomized controlled trial, predominantly focused on academic apps that were not readily available in the commercial market [11]. A few exceptions were observed where commercial software, such as WeChat (Tencent) [22] and WeVideo (WeVideo, Inc) [7], were used. However, these commercial apps were used merely as auxiliary tools, limiting their capacity to support specific phases of the digital storytelling process. Bridging the gap between the lack of empirical proof of app utility and consumer reliance on potentially inaccurate reviews is crucial. An up-to-date and comprehensive review of apps offers an informative and much-needed benchmark in assessing the appropriateness and efficacy of digital storytelling apps, furnishing individuals and caregivers with valuable insights when selecting apps that genuinely cater to the unique needs of those with cognitive impairment. Simultaneously, this research aims to systematically review mobile apps for digital storytelling, evaluating their suitability for people with cognitive impairment. To do so, app quality is evaluated via an expert rating scale, delineating the features of the apps that received the highest scores.

On the basis of the extant body of academic literature [20,23], digital storytelling is conceptualized as comprising 4 components—*capture memory*, *save*, *reminisce*, and *share*—components which can be integrated into 2 prevailing theoretical frameworks: narrative psychology focuses on how individuals construct and understand their experiences through stories, making it particularly relevant for understanding the *capture memory* and *reminisce* functionalities. By contrast, life span developmental psychology emphasizes the importance of preserving memories and social connections at different stages of life, which aligns well with the *save* and *share* functionalities. The decision to use both theoretical approaches is grounded in their complementary nature. While narrative psychology offers insights into the personal and emotional aspects of storytelling, life span developmental psychology provides a broader context for understanding how these activities support cognitive and social well-being across the life span. These frameworks are epistemologically compatible, as both are concerned with understanding human development and behavior through the lens of memory and narrative. By integrating them, we can offer a more holistic understanding of how digital storytelling can be optimized for users with cognitive impairments.

First, narrative psychology studies how individuals construct and understand their experiences through stories. When translated to storytelling apps, this theoretical framework offers great potential for explaining the *capture memory* and *reminisce* functionalities. According to Bruner [24], people form and express their identity by recording and reconstructing their experiences. In digital storytelling apps, the *capture memory* functionality allows users to document life events through photos, videos, and text—transposing a cognitive function into a digital device. This not only helps users construct and understand their stories but also provides rich material for future reminiscence. In addition, narrative psychology emphasizes that individuals can better understand and construct their personal narratives by recalling and reflecting on past experiences [25]. In parallel, the *reminisce* functionality in digital storytelling apps allows users to revisit and reflect on their past, helping strengthen self-identity and promoting emotional health and social interaction [26].

Second, life span developmental psychology is focused on understanding how individuals handle and preserve memories at different life stages. This theoretical framework can correspond to and explain the *save* and *share* functionalities. Significant autobiographical memories shape an individual’s identity, reflect age-related themes of psychosocial development, and contribute to life satisfaction, particularly when these memories are emotionally positive [27]. In digital storytelling apps, the *save* functionality ensures that users can store their memories and stories indefinitely. This is especially important for older adults and individuals suffering from memory-related impairments, as it helps them maintain a sense of continuity in their personal history and narrative as memory declines.

In addition, life span developmental psychology emphasizes the importance of social relationships and emotional support at different life stages. By sharing personal memories and stories, users can enhance social connections and support [22]. The *share* functionality in digital storytelling apps also enables users to share their stories and memories with family and friends, catalyzing social relationships and maintaining social networks, thereby facilitating emotional support—which in turn helps improve psychological health and social well-being [13]. By integrating narrative psychology and life span developmental psychology, we can comprehensively explain the 4 functionalities—*capture memory*, *save*, *reminisce*, and *share*—offered by digital storytelling apps. These functionalities collectively promote cognitive functions, emotional expression, and social interaction, providing older adult-users with greater opportunities for social support, psychosocial betterment, and cognitive recovery. Using this integrated framework not only helps our understanding of the effectiveness of these apps but can also guide future app development to better serve users with cognitive impairments.

### Objectives

This study evaluates the features and quality of digital storytelling apps tailored for individuals with cognitive impairment within the Chinese cultural context. The primary research question focuses on determining the overall effectiveness of these apps in supporting cognitive functions and enhancing user engagement. This study investigates the key features of digital storytelling apps that are designed to support cognitive abilities and stimulate user engagement among a research population of users who are cognitively impaired in China. In addition, the research will investigate how these apps may aid in reminiscence and potentially promote social interactions. Through a systematic analysis of these aspects, the study aims to identify the potential benefits and adaptability of digital storytelling tools in the context of cognitive impairment. The research questions are as follows:

1. What are the features of digital storytelling apps for users who are cognitively impaired?
2. What are the qualities of those apps?
3. What are the users’ recommendations for an effective and engaged digital storytelling app?

## Methods

### Search Process

A systematic search was carried out across China’s Apple Store (Apple Inc) and Google Play (Google LLC). The search encompassed a carefully selected array of keywords in both English and Mandarin, relevant to this study’s focus. These keywords include *storytelling*, *life memories*, *life stories*, *life review*, *reminiscence*, *mild cognitive impairment*, *dementia*, *cognitive impairment,* and *Alzheimer’s disease*. Multimedia Appendix 1 presents the search outcome.

In total, 3 research assistants systematically conducted the searches, downloaded all apps that fell within the inclusion criteria, and independently evaluated each one. For every app, 2 reviewers were assigned to evaluate its relevance; in cases of discrepancy, a thorough discussion ensued to reach a consensus. The Mobile Application Rating Scale (MARS) was then used by the research assistants to assign a score to each screened app.

### App Screening

The inclusion criteria were as follows: (1) a smartphone-based app, (2) compatible with Android or iOS operating systems, (3) the language of the app should be either English or Chinese, (4) the app should have features that contribute to assisted storytelling or reminiscence for those with cognitive impairment, and (5) the app must be available for download in the China app store via iTunes and Google Play.

During the preliminary screening process, the following exclusion criteria were applied, with duplicates removed: (1) app content is merely for information, education, reference, or reading only (ie, no data entry capability); (2) the app only comprises treatment algorithms; (3) the app only supports media editing or saving; (4) original story creation is not supported; and (5) it was explicitly designed for use by clinicians only.

Android apps were downloaded (via the China Google Play store) and tested, capturing memory, saving memory, reminiscence, and sharing features using Huawei P30Pro phones equipped with Android (version 5.1.1; Harmony OS). Similarly, iOS apps were downloaded (via the China iTunes store). Android apps were rated and reviewed using Huawei P30Pro on Harmony OS and Samsung S9 using the Android system. Apple apps were rated and reviewed using iPhone 11 on iOS 13. Each app was tested in a real-world environment for no less than 20 minutes by 3 researchers and scored together after testing was completed. (the PRISMA-ScR checklist is attached in Multimedia Appendix 2).

### Data Collection

A total of 9 digital storytelling apps were evaluated by the research team from December 17, 2023, all of which declared their potential to support individuals with cognitive impairments. During this period, each team member independently documented information gathered from extensive user reviews and feedback available in the Ratings and Reviews sections of the iTunes App store and Google Play platform for each app. The data were extracted and organized using Excel (Microsoft Corp), which allowed for systematic categorization and analysis of user feedback across multiple parameters relevant to our study. This tool facilitated the efficient management and analysis of the large volume of user-generated data. This included fun mental app details (such as its name, country of origin, developers, and user ratings), as well as more in-depth data, such as app description, first launch time, pricing, overall comments, and the number of user comments. Additional data captured include the app’s stated aims, its main features, its target users, user experience, and interaction logic, as well as functionalities related to the cognitive support features of Capture Memory, Save, Reminisce, and Share mentioned earlier. User interface and visual design elements (such as color, imagery, and screenshots) were also recorded and analyzed.

### Data Analysis

Textbox 1 details the analytic methods applied to the collected data; we used a methodological approach designed to focus on different aspects of digital storytelling apps. Thematic analysis was used to identify and report patterns or themes within the description of apps, summarizing key features and evaluating commonalities across apps [28]. Similarly, thematic analysis was used to categorize and interpret platform diversity, geographic origins of app developers, and app ratings, helping to ascertain the spread and focus areas of app development across different regions and platforms. Thematic was then used to systematically categorize specific features of digital storytelling apps, allowing a structured comparison of more nuanced functionalities, such as *capture memory*, *save*, *reminisce*, and *share*, highlighting how these features support cognitive functions and user engagement. Statistical analysis was conducted based on the MARS [29] ratings to evaluate app quality. Mean scores were calculated across 4 domains: engagement, functionality, esthetics, and information quality. MARS is a validated tool widely used in other studies to assess mobile health apps. MARS is structured as a Likert-type scale, with items rated from 1 (inadequate) to 5 (excellent) across 4 domains: engagement, functionality, aesthetics, and information quality [29]. MARS showed strong internal consistency and interrater reliability, offering a dependable approach for rating and comparing mobile apps [30,31].

**Data collected and type of analysis used.**

**Analysis and data collected**

- Thematic analysis
  Description of apps: platform diversity, geographical distribution of develope, and features of digital storytelling apps
  User feedback and engagement
- Descriptive statistics
  App rating and quality assessment of digital storytelling apps: Mobile Application Rating Scale rating

All collected data were collaboratively reviewed by the 3 research assistants to ensure accuracy, consistency, and impartiality in the final evaluation. The data analysis process was structured to ensure a comprehensive and accurate evaluation of the apps. Initially, key areas for app development and improvement were highlighted through data summarization using Excel, where the research team organized and extracted essential data points from the app stores. Following this, each research assistant independently analyzed the data, assessing the apps’ features and performance.

The team engaged in group discussions to review the independently analyzed data to ensure consistency and minimize individual biases. Discrepancies were resolved through these discussions, leading to a consensus on the final data extraction. This collaborative approach not only enhanced the reliability of the findings by incorporating diverse perspectives but also provided a foundation for identifying areas where the apps could be further developed and improved.

The research team used a detailed coding framework to systematically categorize feedback and MARS scores, ensuring a thorough and structured analysis. Regular cross-checks and validation steps were integrated throughout the review process to maintain data integrity and validity. Periodic meetings were also held to discuss emerging themes and refine the analysis methodology where necessary. This rigorous review process not only ensured the accuracy and consistency of the final evaluation, but also yielded comprehensive insights into the strengths and weaknesses of each app, thereby informing targeted recommendations for future app development vis-à-vis users who are cognitively impaired.

## Results

### Search Outcome

**Figure 1:**
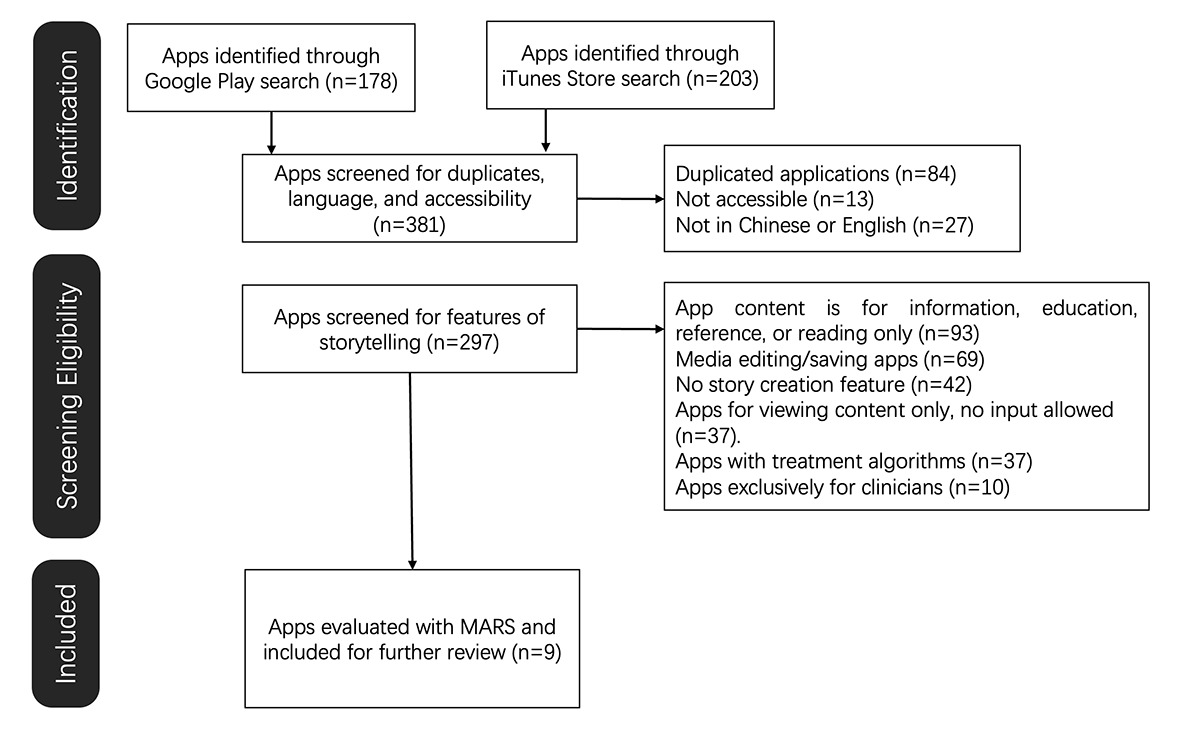
Flow diagram of systematic search and selection of apps from the Google Play and iTunes stores. MARS: Mobile Application Rating Scale.

As depicted in Figure 1, Google Play yielded 46.7% (178/381) apps, while iTunes had a slightly higher yield with 53.3% (203/381) apps. After removing duplicates, the aggregate number of unique apps stood at 77.9% (297/381). The screening process then ensued, during which 4.3% (13/297) apps were found to be inaccessible; another 9.1% (27/297) apps did not offer Chinese or English language options. This filtration culminated in the inclusion of 9 apps that met all the stipulated criteria for further consideration.

### Overview of Digital Storytelling Apps

Table 1 provides an overview of the included storytelling and memory apps, detailing their platform compatibility, developer origins, user ratings, and feedback across both iOS and Android platforms.

**Table 1:** Overview of digital storytelling apps.

App name	Description	Country	Platform	Rating	Pricing	Number of comments
Story Dice: Story Telling	Game with 40 dice, 240 images, and unlimited stories. Mixed categories such as mystery or Star Wars.	Netherlands	Both iOS and Android	iOS: 4.6 and Android: 4.5	Free	iOS: 8 and Android: 0
Grand Storyteller	Create and share multimedia stories and convert stories into videos.	United States	iOS	No score provided	Free	—^a^
Memoirs of Life	Record daily life with documents, labels, and stickers. Add and edit photos.	China	Android	4	Free with membership	—
Spicy Memories	AI^b^ selects and crops photos, sets time ranges, and shares photos as wallpapers or collages.	China	iOS	4.6	Free	245
PWI Storyteller	Share stories with nonprofits to help them tell their stories more effectively.	United States	iOS	No score provided	Free	—
Brief Memories of Photos	Simple Photo app groups photos with labels for easy retrieval.	China	iOS	3.6	Free	5
FamilySearch	Preserve and share family memories, create digital scrapbooks, capture important moments, identify relatives in photos, and sync across devices.	United States	Both iOS and Android	iOS: 4.9 and Android: 5.0	Free	iOS: 234 and Android: 47,400
Reminisce	Trivia game on American pop culture with categories such as television shows, movies, sports, history, and commercials.	China	Android	—	Free	—
Memorize: Diaries, Memories, Notes, Ideas, Timelines, Categories	Diary app for recording memories, thoughts, and events. Features include adding photos, voice notes, PIN^c^ or fingerprint security, multiple themes, and backup or restore.	Unknown	Android	3.8	Free	3892

^a^Not applicable.

^b^AI: artificial intelligence.

^c^PIN: personal identification number.

All the apps included in our analysis are available (at least initially) for free, a common strategy used to increase user initial engagement without requiring a financial commitment. Only Memories of Life has a paid membership feature, which is necessary for users to export memories. Another shared aspect is their focus on storytelling or memory recording. This indicates a targeted market interest: users who are keen on documenting personal experiences, storytelling, or creatively engaging with content over periods. All these apps aim to preserve and share personal memories, stories, and experiences in a creative and engaging manner. They provide tools for users to manually record, organize, and share content, such as text, images, audio, and video. By and large, the apps are designed to be user-friendly and offer features such as tagging, categorizing, and integrating with other platforms for seamless sharing and retrieval of memories. They also emphasize personalization, enabling users to add their unique touches to their memories and stories.

Story Telling and Grand Storyteller, both encourage creative storytelling. The Story Telling app highlights its function of randomly displaying images, which prompts users to create imaginative stories. Story Telling uses dice with images to inspire the creation of fictional stories, encouraging users to craft narratives that are entirely imaginative and creative. The app’s design promotes storytelling as a fun and interactive family activity, where participants use the random images provided by the dice to generate unique and inventive fictional stories. Grand Storyteller encourages detailed multimedia stories with text, images, audio, and video, which is perfect for documenting personal experiences and creating personalized audio storybooks. Memoirs of Life and Memorize both serve as digital diaries. Memoirs of Life is a simple app for documenting daily activities, moods, and thoughts with stickers and photos, whereas Memorize offers more features, including secure detailed memory recording with photos, voice notes, categories, tags, and themes, catering to a wide range of diary-keeping needs. Spicy Memories and Brief Memories of Photos function more like tools for organizing and curating photos.

Spicy Memories uses artificial intelligence (AI) to help users select and crop photos for rediscovery and sharing as wallpapers or photo collages. Brief Memories of Photos allows users to label photos for easy searching and revisiting, helping users efficiently manage photo collections. PWI Storyteller offers an innovative approach by connecting users with nonprofits, enabling them to share personal stories that organizations can use to promote their causes and engage audiences, fostering meaningful connections for social impact. FamilySearch helps preserve and share family memories by creating digital scrapbooks of important moments, enabling integration with other platforms for ease of access and sharing, making it ideal for documenting family heritage. The Reminisce app incorporates cultural elements by offering predefined story prompts that reflect various aspects of American pop culture, including African American heritage. This feature allows users to engage with culturally relevant content, which could include trivia about television shows, movies, sports, and historical events. In addition, users have the option to create their own stories, which provides a way to personalize their experience further. By integrating these elements, the app not only offers an engaging and educational platform but also allows users to explore and connect with cultural memories, potentially using their own photos to enhance and personalize the storytelling process.

### Platform Diversity, Geographical Distribution of Developers, and App Ratings

The 9 apps sampled in this research exhibit significant diversity in terms of platform availability. About 44% (4/9) of the apps are exclusive to iOS, reflecting a preference (or perhaps a strategic decision) to target Apple’s ecosystem, which is known for its more controlled user experience. In contrast, 22% (2/9) are exclusive to Android, which may cater to a broader user base given Android’s larger global market share. Another 22% (2/9) are available on both iOS and Android, ensuring the widest possible accessibility. About 11% (1/9) of the total app’s platform is unknown.

The geographical distribution of the app developers also exhibits significant variance, with China producing 33% (3/9) of the apps, reflecting significant input from Chinese developers in the app market. The United States is responsible for 22% (2/9) of the apps, demonstrating its continued influence in the global tech scene. About 11% (1/9) comes from the Netherlands, and the remaining 33% (3/9) have developers listed as global or unknown. All in all, this composition reflects a range of diverse or multinational development efforts.

About 67% (6/9) of the apps have a user rating of 4.0 or above, suggesting generally positive user feedback (where ratings are provided). Furthermore, 22% (2/9) of the apps have a high level of user engagement, with >200 comments on their respective platforms, highlighting significant interactive participation by users with those apps.

### Features of Digital Storytelling Apps

The 9 sampled apps boast 4 principal functionalities: *capture memory*, *save*, *reminisce*, and *share* (Table 2). The *capture memory* feature allows users to take notes, snap pictures, or record videos directly through their smartphones, as well as upload existing media. The *save* function ensures that stories are not only displayed but also preserved for future access, transcending ephemeral experiences. *Reminisce* leverages these materials to facilitate the recollection of memories, enriching the user’s reflective experience, such as via playback functionalities. Finally, *share* extends the reach of users’ stories, enabling them to connect with a broader audience through digital or social media sharing. All apps incorporate reminiscence and outputting stories in the form of words or pictures.

**Table 2:** Comparative features of storytelling apps: capturing, saving, reminiscing, and sharing memories.

App	Capture memory	Save	Reminiscence	Share
Story Dice: Story Telling			✓^a^	
Grand Storyteller	✓	✓	✓	✓
Memoirs of Life		✓	✓	✓
Spicy Memories		✓	✓	
PWI Storyteller	✓	✓	✓	✓
Brief Memories of Photos		✓	✓	✓
FamilySearch	✓	✓	✓	✓
Reminisce			✓	
Memorize: Diaries, Memories, Notes, Ideas, Timelines, Categories	✓	✓	✓	✓

^a^Supported the features from the app.

Most apps (7/9, 78%) offer saving memories or stories, and 67% (6/9) apps support sharing the stories. About 22% (2/9) apps did not have *store memory stories* (Story Dice: Story Telling, and Reminisce), and 33% (3/9) did not have user tips (Grand Storyteller, Memoirs of Life, and Memorize: Diaries, Memories, Notes, Ideas, Timelines, Categories). Around 44% (4/9) apps provided memory themes (Grand Storyteller, PWI Storyteller, FamilySearch, and Reminisce). Grand Storyteller was unique in that it offered some broader story themes, which are illustrated by a cartoon picture and a sentence. PWI Storyteller is classified by some specific items or activities (potentially diseases for older adults-users) to determine the theme of the story. FamilySearch automatically determines the theme of memory through some very detailed guiding questions, whereby user answers influence the automated formation of stories. In *reminisce*, under each memory theme, an actual photo is in the foreground and questions about important life events are asked.

Table 3 synthesizes the findings from our analysis of the storytelling apps. The main columns in the table were created through thematic analysis of the features and functionalities identified across the apps. The “app community” column refers to the social features within each app that allow users to share their memory stories with others, fostering a sense of community. Memory Theme represents the categorization or thematic organization of the stories, which may be guided by the apps to help users focus on specific types of memories, such as childhood or significant life events. Classification denotes how the apps organize the content, whether by media type (text, photo, and video) or by chronological or thematic order. Usage Tips refers to the guidance or suggestions provided by the apps to enhance user engagement, such as best practices for creating and sharing stories or ways to use the app more effectively. These columns capture the core features shared across the storytelling apps. For instance, all the apps require users to upload and edit their memory-derived stories, providing materials for engaging in reminiscence. In addition, 89% (8/9) apps allow users to present their stories in multiple formats, such as text, photos, or videos.

**Table 3:** Shared features across storytelling apps.

App	App community	Memory theme	Classification	Use tips
Story Dice: Story Telling				✓^a^
Grand Storyteller		✓		
Memoirs of Life	✓		✓	
Spicy Memories			✓	✓
PWI Storyteller	✓	✓		✓
Brief Memories of Photos			✓	✓
FamilySearch		✓	✓	✓
Reminisce		✓		✓
Memorize: Diaries, Memories, Notes, Ideas, Timelines, Categories			✓	

^a^Supported the features from the app.

First, it can be seen that these storytelling apps all require users to upload their own memory-derived stories and use their own memories to edit and generate stories, providing materials for users to engage in reminiscence. Users can use the apps to browse through their own memory stories. Second, when using these apps (8/9, 89%), users can usually choose to present their memory stories in text, photos, or video form.

When engaged in creating their own memory stories, different apps use different methods for selecting the story’s theme. For instance, Story Dice inspires users’ creativity by letting them roll the dice in the app to create fictional stories, allowing users to invent characters, settings, and plots based on the outcomes of the dice rolls. Pela Memory uses AI technology to automatically select and crop photos uploaded by users to create collages. This kind of story generating method reduces the user’s learning cost (which may be better suited for older adults who are cognitively impaired) and can guide users to pay attention to details in their photos that they have never noticed before, making user engagement with memory stories more fun and potentially aiding memory recall. In addition, these apps also differ in how they can classify user memories. There are 2 main ways of classification. One is to enable users to add tags themselves to classify their memories, whereby users edit memory classification tags manually, making it more flexible when using this kind of storytelling app. The other is that the app itself already has classifications, and users only need to add their own stories or photos under the preexisting classifications. This second approach may be more convenient and easier for cognitively impaired users to learn.

### Quality Assessment of Digital Storytelling Apps: MARS Rating

To provide a systematic quality assessment, our research evaluated a sample of the available storytelling apps using the MARS across 4 domains: engagement, functionality, aesthetics, and information, culminating in an overall score (Table 4). The range of the mean score of MARS is 2.33 to 3.35, (SD 0.60). The reliability of the MARS was assessed using intraclass correlation coefficients (ICCs) across various categories. The results indicate that information quality demonstrated the highest reliability with an ICC of 0.81, suggesting substantial agreement among raters. Similarly, overall app quality also showed substantial reliability with an ICC of 0.71. The engagement category had moderate reliability with an ICC of 0.49, while the functionality and aesthetics categories exhibited fair reliability with ICCs of 0.37 and 0.38, respectively. The evaluation of digital storytelling apps using MARS yielded varied results across the 9 apps sampled, with scores reflecting different strengths and weaknesses regarding engagement, functionality, esthetics, and information quality. Memoirs of Life obtained a score of 3.6, 3.75, 3.3, and 2.4 over each of the domains, respectively, with a mean score of 2.33. Memorize: Diaries, Memories, Notes, Ideas, Timelines, Categories exhibited a slightly more consistent performance across domains, with scores of 3.4, 3, 3, and 2.9 (respectively), resulting in a mean of 3.06. Grand Storyteller emerged as a stronger contender for use by older adults who are cognitively impaired, with scores of 3.8, 3.75, 3.3, and 2.3 (respectively) and a mean score of 3.35. Spicy Memories and PWI Storyteller were found to be midrange, with mean scores of 3.18 and 3.05, respectively. Brief Memories of Photos and FamilySearch both demonstrated modest scores across all 4 domains, with a mean of 3.02 and 3.17 (respectively). Reminisce and Story Dice: Story Telling rounded out the evaluation, recording mean scores of 3.10 and 3.04 (respectively). The outcome of this assessment demonstrates the diverse capabilities and varying quality of digital storytelling apps currently available, reflecting a broad spectrum of user experiences and satisfaction levels.

**Table 4:** Average Mobile Application Rating Scale rating.

App	Engagement	Functionality	Aesthetics	Information	Scores, mean (SD)
Memoirs of Life	3.6	3.75	3.3	2.4	2.33 (0.77)
Memorize: Diaries, Memories, Notes, Ideas, Timelines, Categories	3.4	3	3	2.9	3.06 (0.70)
Grand Storyteller	3.8	3.75	3.3	2.3	3.35 (0.60)
Spicy Memories	3.4	3.5	3.7	2	3.18 (0.78)
PWI Storyteller	3	3.5	3.3	2.3	3.05 (0.53)
Brief Memories of Photos	3.2	3.25	3	2.3	3.02 (0.44)
FamilySearch	3.2	3.75	3.3	2.3	3.17 (0.61)
Reminisce	3.4	3.25	3.3	2.4	3.10 (0.46)
Story Dice: Story Telling	3	3.5	3	1.7	3.04 (0.22)

### User Feedback and Engagement

User feedback and engagement across the digital storytelling apps reveal a diverse range of experiences and sentiments, which can be grouped into several key themes.

#### Overview of Comments

Despite the diverse range of comments, the overall comment across these apps is slightly positive, with 5231 positive reviews compared with 4280 negative ones. User feedback also showed considerable variability, ranging from 0 comments for apps such as Grand Storyteller and PWI Storyteller to as many as 5361 comments for FamilySearch, which received extensive positive reviews. This suggests a generally mediocre reception, with some apps, such as FamilySearch and Memorize, receiving high praise for their functionalities, while others, such as Spicy Memories, show room for improvement, particularly in user interface and flexibility. The diversity in feedback highlights the importance of continuous updates and improvements to meet user needs and enhance satisfaction, particularly for cognitively impaired users.

#### Ease of Use and User Interface

Users frequently mentioned the importance of a user-friendly interface. For example, Story Dice: Story Telling was generally found enjoyable, particularly for both adult and child users. However, criticisms arose regarding advertisements that could not be closed, which detracted from the overall user experience and could potentially confuse cognitively impaired users. Similarly, Spicy Memories faced criticism for becoming less user-friendly over time, as updates made the app more restrictive in editing options.

#### Content and Feature Satisfaction

Several apps received praise for specific features that enhance the user experience. FamilySearch garnered extensive positive feedback, with users appreciating its ability to collect and preserve family memories by adding photos, documents, and recordings to their family tree. This app also had the highest number of comments, indicating significant user engagement. By contrast, Brief Memories of Photos was highlighted for its effective tagging and organizing functionalities, though more detailed feedback was scarce.

#### Customization and Flexibility

Customization options were a point of contention for users. While Spicy Memories was praised for its innovative AI-driven features, such as rediscovering old photos and photo cropping options, users expressed dissatisfaction with the app’s growing restrictions, reflecting challenges in balancing feature development with user flexibility. In addition, Memorize: Diaries, Memories, Notes, Ideas, Timelines, Categories received generally favorable reviews, particularly for its diary and note-taking functions, but users suggested improvements, such as enhanced data backup options, better security settings, and additional synchronization options.

#### Market Penetration and User Engagement

The level of user engagement varied widely among the apps. Grand Storyteller, PWI Storyteller, and Reminisce had no user comments available, which could indicate either low user engagement or limited market penetration. Memoirs of Life also lacked specific user feedback, making it challenging to assess user satisfaction or identify areas for improvement. The scarcity of comments for these apps suggests a relatively small target audience or recent entry into the market.

## Discussion

### Principal Findings

The principal findings from the evaluation of 9 digital storytelling apps using the MARS offer significant insights into the app landscape. Among the apps evaluated, Memoirs of Life and Memorize: Diaries, Memories, Notes, Ideas, Timelines, Categories scored the highest, with Grand Storyteller also performing well, indicating a balance in user engagement, functionality, esthetics, and information quality. Story Dice: Story Telling yielded the lowest mean score at 3.04, primarily due to its deficiencies in information quality. Apps with lower information quality scores are likely to struggle with user retention and credibility, which are critical for maintaining user interest. Users’ feedback on specific app features and innovative features such as the photo classification functionality received positive feedback, contrasting with criticisms of Story Dice for its lack of innovation.

Apps, such as FamilySearch, have received extensive positive feedback and high user engagement, whereas others, such as Grand Storyteller, have received minimal feedback, highlighting market penetration inconsistencies and varying user interest levels. The geographical distribution of app developers, with significant contributions from China and the United States, indicates a global interest in digital storytelling technologies.

In summary, while certain apps set benchmarks for excellence in the digital storytelling domain, others lag, particularly in crucial areas, such as information quality. This disparity accentuates the need for ongoing improvements in app functionality and user interface to better cater to specific user groups, particularly those with cognitive impairments.

### Features of Storytelling Apps

Traditional photo organization features on mobile devices often fall short of more complex or customizable sorting, making the structured and intuitive classification functions provided by storytelling apps highly beneficial [32,33]. These apps simplify the management of photos, offering a supportive user experience for the cognitively impaired. In addition, some apps incorporate AI to automatically generate photo albums and recall key life milestones, which can further support users in selecting appropriate materials for reminiscence [8]. This innovative use of technology simplifies data interaction as well as encourages sharing memories within social groups, which can improve social relationships and foster new connections, especially among older adults [34]. The effective use of AI and organization features in storytelling apps plays a crucial role in supporting cognitive functions and enhancing social engagement among users. AI can perform an automated role in curating, summarizing, linking, and presenting vast amounts of image data in a manner that is particularly helpful for the cognitively impaired. In this context, storytelling emerges as a fitting metaphor, adept at capturing and illustrating the narratives and insights hidden within the relationships among data scattered across various repositories. The difference, however, lies in the fact that commercial apps have introduced AI for automatic filtering and reminders.

In addition, helping users to share their memories or stories in a group by offering preset themes is also one of the ways to increase positive user engagement and, by extension, positive emotions. Such an approach is not only innovative but also instrumental in making complex data interactions more accessible and engaging for users, especially for older adults or those who are cognitively impaired. This underscores the untapped potential of commercial apps that could leverage AI to transform how we interact with and interpret image data, turning it into compelling and informative digital narratives that are particularly valuable in enhancing cognitive engagement among older adults. This form of sharing in a group, by contrast, can also support users in maintaining or enhancing their social relationships and making new social connections. Academic research has shown that the choice of story themes is limited, and there is little support for group activities [6,7], revealing a research and operational gap. Finally, the sample analyzed herein revealed how some apps focus on the entertainment value they provide. These apps often achieve this through some special interaction or interactive logic. For instance, by shaking the phone to simulate a dice roll, the app reads the contents of a photo album to automatically generate memories or to locate people nearby who have similar experiences, thereby encouraging face-to-face meetings or walks together.

### Quality of Digital Storytelling Apps

The quality of these apps, as assessed using established methods such as the MARS, shows considerable variation. For instance, apps such as Memoirs of Life and Memorize: Diaries, Memories, Notes, Ideas, Timelines, Categories rank highly due to their comprehensive features and user-friendly interfaces, all of which are conducive to supporting cognitive functions [32,33]. In contrast, other apps may have lower scores due to less effective implementation of features or poorer information quality, aspects that are crucial for users who rely on accurate reminiscence triggers and social interaction support [35-37]. The effectiveness of digital storytelling apps, particularly within the Chinese cultural context and for individuals with cognitive impairments, such as dementia, reveals a high degree of potential yet varied outcomes [4]. This statistical approach provided a quantifiable measure of app utility and quality, facilitating objective comparisons. Thematic analysis was additionally used to gauge user feedback and engagement levels, a process that entailed an examination of user reviews to identify common sentiments, areas of satisfaction, and points of contention. This layer of analysis helps us to understand the user experience and the impact of app updates on user satisfaction.

These apps, which aim to engage users—especially older adults—often suffer from a gap in quality and a lack of specialized features. This results in an overall scarcity of intuitive and cognitively engaging apps, as is demonstrated by the limited user comments and a lack of engagement. These factors suggest a need for apps more tailored to specific user needs. Moreover, there is a clear oversight in the accessibility and practicality of these apps for public use, highlighting an urgent need for focused development and rigorous evaluation to enhance their relevance and effectiveness in promoting cognitive engagement. This finding is supported by various studies that demonstrate the benefits of digital reminiscence therapy, which can facilitate significant enhancements in cognitive function and emotional well-being among participants. Studies by Moon and Park [38], Mulvenna et al [39], Derbring et al [40], and Sarne-Fleischmann et al [41] illustrate how these interventions not only improve engagement and interaction among older adults and caregivers but also emphasize the importance of personalized digital systems in improving recall abilities among older adults who are cognitively impaired, especially among older adults with Alzheimer. Collectively, these findings provide an empirical basis to support the continued development and integration of digital storytelling tools in therapeutic and care settings, highlighting the potential of these technologies to significantly improve cognitive functions, emotional well-being, and interpersonal interactions, thereby enhancing the overall quality of life for cognitively impaired users. While this research has filled a gap in the academic literature, it has also revealed an important gap between empirical findings and commercially available solutions for the cognitively impaired.

### Recommendations for Effective and Engaged Digital Storytelling

Synthesizing the user feedback indicates that there is a need for more interactive and personalized features in digital storytelling apps to improve engagement and effectiveness in cognitive and social support. Digital storytelling apps facilitate reminiscence and social interaction among users with cognitive impairment by providing functions that are both engaging and relatively easy to navigate. For instance, apps such as FamilySearch and Memoirs of Life offer functionalities that not only allow users to store and recall memories but also share these with others. This sharing capability is advantageous and yields therapeutic benefits, as it enables users to connect with family and friends, fostering social interactions that are vital for mental health and social well-being. Moreover, the integration of AI technologies in apps such as Spicy Memories enhances the curation and presentation of personal content, making it more accessible and engaging for users; this not only supports cognitive engagement through interactive reminiscence but also makes the apps easier to use and thus less burdensome for the cognitively impaired. Digital memory books that incorporate personal photos, videos, and narratives can improve the emotional and social well-being of older adults.

The strategies of these apps in terms of functionality are almost identical and offer little differentiation. For instance, some apps allow memories to be cataloged according to life events or specific topics, providing a structured approach (ie, timeline) to storytelling [11]. Moreover, there are apps that index memories by locations and dates, offering strategies to track recollections over time. These features enhance the user experience by simplifying the process of memory retrieval and organization, aiding in memory recall. In addition to the timeline organization strategy used in the reminiscence phase of digital storytelling, these apps offer significant potential for the integration of other stages and behavioral change strategies. These include setting behavior goals and outcome objectives, which can play a crucial role in structuring the storytelling process [42] and encouraging cognitive exercises. Specifically, in the reminiscence phase, the introduction of scaffolding techniques can significantly aid older users in recalling memories [43]. This could involve the use of guided questions or prompts that help in fleshing out stories more comprehensively. By incorporating such strategies, digital storytelling apps can become more than just tools for memory capture; they can actively assist users in weaving richer, more detailed narratives, thereby enhancing the overall storytelling experience for older adults.

Regarding story-sharing functionalities, in academic settings, sharing is found to be more oriented, toward family members and participants who are involved, which is understandable given the personal nature of photographs [14,44]. Narrating personal experiences and events offers a wealth of subjects for discussion and chances to establish connections with others [45]. Discussing our own life experiences is recognized as integral to forming relationships and is a primary method through which we pursue and establish connections with others. In this research, we observed distinct technical features across various storytelling apps, each designed to enhance the user experience in unique ways. Memoirs of Life stands out with its sharing capability, necessitates user login, and requires internet access for its functionality. PWI Storyteller similarly offers a sharing feature that requires internet access. A notable aspect of FamilySearch is its facilitation of family members sharing a common database, with the added advantage of memory sharing through networking. This app seamlessly synchronizes with FamilySearch, which facilitates a fluid experience across different devices by enabling users to pick up where they left off (a reduction in the necessary steps, thereby increasing accessibility for the cognitively impaired). Memorize offers a robust solution for journaling, with features for permanent storage, backup to Google’s cloud storage, and the ability to import and export memories in different formats, including ZIP files. By contrast, some apps did not exhibit specific technical features.

Commercial apps offer several advantages in supporting story sharing, particularly for older adults or cognitively impaired users. A promising feature is the ability to invite family members to collaborate and edit stories together, fostering a sense of community and intergenerational connection [45]. Furthermore, research into the mediating effects of internet use reveals that it enhances the propensity of older adults to engage with their communities, partly due to the improvement in their subjective health perceptions [46]. By providing an easy and accessible platform for sharing personal narratives, commercial storytelling apps not only promote social interaction but also potentially improve the overall well-being of older adults, which is conducive to psychosocial and cognitive therapy.

### Research Implications and Future Directions

This research identifies a need for more comprehensive studies that not only explore commercially available digital storytelling tools but also examine their adaptability and relevance for older and cognitively impaired users. Such studies should aim to address the usability challenges these apps often present and explore how AI-enhanced features can further support cognitive functioning, mental health, and social well-being. By focusing on these aspects, future research can significantly contribute to the development of digital storytelling apps that are more inclusive, effective, and tailored to meet the unique needs of this population segment. Ongoing research is needed to further explore the long-term effects of digital storytelling apps, the best practices for their design and usability, and how they can be integrated into broader dementia care strategies.

### Strength and Limitations

This study offers an initial exploratory review of digital storytelling apps, assessing their quality and suitability through a newly developed multidimensional expert rating scale. This scale provides a comprehensive measure of app quality across 4 objective domains (engagement, functionality, aesthetics, and information quality) and incorporates a subjective aspect. These objective quality dimensions, which contribute to the overall app quality score, offer a quantitative means for comparative analysis and can set a benchmark for future research.

Nonetheless, this study has a few limitations. First, while the rating scale is effective in evaluating the current milieu of apps, it cannot replace the need for user-centered design and evidence-based practices in app development, especially within the health behavior sector. A significant limitation of this study is its geographic restriction to the Chinese market, meaning that apps that may be available in other regions have not been considered. This limitation may have resulted in the exclusion of potentially relevant apps that are not accessible within the Chinese market, thus limiting the scope of this review. Future research would do well to increase the geographic scope of analysis. Future studies should also focus on evaluating specific storytelling features and guided content within these apps, as there is currently no established standard for optimal storytelling practices or their efficacy in mitigating symptoms of cognitive impairment. This study did not establish a standard for optimal storytelling practices, particularly regarding the use of fictional versus reminiscence-based storytelling in digital apps for older adults. The differences between these 2 types of storytelling and their respective impacts on cognitive function remain underexplored. Future research should aim to delineate the cognitive benefits of each storytelling approach, particularly for older adults with cognitive decline.

### Conclusions

This systematic search and evaluation of digital storytelling apps in the Chinese app ecosystem reveals a gap in the quality and features of these apps, particularly for users with cognitive impairment. Our findings, derived from an extensive search in both English and Chinese on the Google Play Store and iTunes Store, identified 297 unique apps, with only 9 (3%) meeting our criteria for further evaluation. The apps sampled herein and evaluated using MARS, exhibited varied levels of quality. Key functionalities such as capture memory, save, reminisce, and share were not uniformly present across all apps. The highest-rated apps were Memoirs of Life and Memorize: Diaries, Memories, Notes, Ideas, Timelines, Categories, indicating a balance in engagement, functionality, and information quality. However, overall, many apps reveal significant room for improvement, especially in the information domain (where user complaints were concentrated). This exploratory study lays the foundation for future research and development in digital storytelling apps, aiming to enhance user experience and effectiveness. The significance of these results lies in guiding the development of more effective digital storytelling apps to maximize their therapeutic potential with a focus on the cognitively impaired. In parallel, the findings emphasize the need for improved content accuracy, user interface design, and the integration of AI technologies to enhance cognitive support and user engagement by aiding in photo restoration and material selection.

## Appendix

Multimedia Appendix 1 Search outcome.

Multimedia Appendix 2 PRISMA-ScR (Preferred Reporting Items for Systematic Reviews and Meta-Analyses Extension for Scoping Reviews) checklist.

## Abbreviations

AI: artificial intelligence
ICC: intraclass correlation coefficient
MARS: Mobile Application Rating Scale
