# Supplementary material for: Digital Storytelling for People With Cognitive Impairment Using Available Mobile Apps: Systematic Search in App Stores and Content Analysis
Source: JMIR Aging. 2024 Oct 24;7:e64525. doi: 10.2196/64525 (PMC11544346; doi:10.2196/64525)
Supplement: Multimedia Appendix 2 [file aging_v7i1e64525_app2.docx]

**Preferred Reporting Items for Systematic reviews and Meta-Analyses extension for Scoping Reviews (PRISMA-ScR) Checklist**

| **SECTION** | **ITEM** | **PRISMA-ScR CHECKLIST ITEM** | **REPORTED ON PAGE #** |
| --- | --- | --- | --- |
| **TITLE** | | | |
| Title | 1 | Digital Storytelling for People with Cognitive Impairment: A Review of Available Mobile Apps. | 1 |
| **ABSTRACT** | | | |
| Structured summary | 2 | Background: A growing body of evidence suggests significant cognitive and social health benefits can be derived from digital storytelling for older adults with cognitive impairment. Digital storytelling applications offer the potential to serve as an on-demand, easy-to-access platform for enhancing cognitive abilities and promoting social well-being. Yet while the quantity of such apps available on the market has increased, there is a pertinent gap in research investigating their quality.  Objective: The objective of this study is to conduct a systematic review of digital storytelling apps available in the Chinese market, evaluating them in accordance with the Mobile Application Rating Scale (MARS). The goal is to identify key features and evaluate the overall quality of these apps in the context of cognitively-impaired users, filling an important research gap.  Methods: A systematic search was conducted in both the Google Play Store and iTunes Store, using English and Chinese languages. Apps were chosen according to specific criteria that included features, including (but not limited to) memory capture, story saving, cue-based reminiscing, and the ability to share stories or memories with others. The Mobile Application Rating Scale (MARS) was employed by three individual researchers to independently assess app quality across a number of domains, such as engagement, functionality, aesthetics, and information quality, for both Android and iOS applications.  Results: From an initial screening of 297 apps, only 9 met the criteria for detailed evaluation using MARS. The reviewed apps featured ‘Capture Memory’, ‘Save’, ‘Reminisce’ and ‘Share’ functions, which are critical in supporting cognitive functions and enhancing user engagement. The analysis revealed notable patterns in platform diversity and geographic distribution of developers, with apps available on both iOS and Android and developers concentrated in China and the USA. ‘Memoirs of Life’ and ‘Memorize: Diaries, Memories, Notes, Ideas, Timelines, Categories’ had the highest mean MARS scores of 3.35, reflecting strong engagement, functionality, and information quality. However, the overall mean score across all apps was only 3.03, reflecting considerable variation, particularly in information quality. User feedback also varied widely, with apps like ‘FamilySearch’ receiving extensive positive reviews and a high volume of comments (5,361 comments across platforms), while others, such as ‘Grand Storyteller’ and ‘PWI Storyteller’ exhibited minimal user engagement. This variability in user feedback underscores the need for continuous improvement and user-centered design, especially the need for improvements in information quality and content accuracy, to enhance the effectiveness of digital storytelling applications to better meet the needs of cognitively-impaired users.  Conclusions: The systematic search and evaluation highlight the diverse capabilities yet variable quality of digital storytelling apps available within the Chinese market, reflecting user experiences, satisfaction levels, and ultimately, efficacy in supporting cognitively-impaired users. While some apps excel in engagement and functionality, others need significant improvements in information quality and user interface design to better serve those with cognitive impairments. Future research is recommended to investigate regional limitations, as well as features which would result in more inclusive and effective digital storytelling applications. | 1 |
| **INTRODUCTION** | | | |
| Rationale | 3 | This present study evaluates the features and quality of digital storytelling applications tailored for individuals with cognitive impairment within the Chinese cultural context. The primary research question focuses on determining the overall effectiveness of these applications in supporting cognitive functions and enhancing user engagement. This study investigates the key features of digital storytelling applications that are designed to support cognitive abilities and stimulate user engagement among a research population of cognitively-impaired users in China. Additionally, the research shall explore how these applications aid in reminiscence, and their promotion of social interactions. Through a systematic analysis of these aspects, the study aims to identify the potential benefits and adaptability of digital storytelling tools in the context of cognitive impairment. | 4 |
| Objectives | 4 | The research questions are as follows:  1. What are the features of digital storytelling apps for cognitively-impaired users?  2. What are the qualities of those apps?  3. What are the users’ recommendations for an effective and engaged digital storytelling app? | 5 |
| **METHODS** | | | |
| Protocol and registration | 5 | Not applicable but checked that no similar review research exist. | Not applicable |
| Eligibility criteria | 6 | The inclusion criteria are as follows: 1) a smartphone-based app; 2) compatible with Android and/or iOS operating systems; 3) the language of the app should be either English and/or Chinese; 4) the app should have features which contribute to assisted storytelling or reminiscence for those with cognitive impairment; 5) the app must be available for download in the China app store via iTunes and/or Google Play.  During the preliminary screening process, the following exclusion criteria were applied, with duplicates removed: 1) app content is merely for information, education, reference, or reading only (i.e., no data entry capability); 2) the app only comprises treatment algorithms; 3) the app only supports media editing or saving; 4) original story creation is not supported; 5) it was explicitly designed for use by clinicians only. and publication status), and provide a rationale. | 5 |
| Information sources* | 7 | Android apps were downloaded (via the China Google Play store) and tested. iOS apps were downloaded (via the China iTunes store). | 6 |
| Search | 8 | A systematic search was carried out across China’s Apple Store and Google Play. The search encompassed a carefully-selected array of keywords, in both English and Mandarin, relevant to this study’s focus. These keywords, including ‘storytelling (讲故事)’, ‘life memories (人生回忆)’, ‘life stories (人生故事)’, ‘life review (回忆录)’, ‘reminiscence (回忆，怀旧，回想)’, ‘mild cognitive impairment (轻度认知障碍)’, ‘dementia (痴呆)’, ‘cognitive impairment (认知症)’ and ‘Alzheimer’s disease (阿尔兹海默症)’. See Multimedia Appendix 1 for the search outcome. | 5 |
| Selection of sources of evidence† | 9 | Android applications were rated and reviewed using HuaWei P30Pro on Harmony OS and Samsung S9 using the Android system. Apple applications were rated and reviewed using iPhone11 on iOS 13. Each application was tested in a real-world environment for no less than 20 minutes by three researchers, and scored together after testing was completed. | 6 |
| Data charting process‡ | 10 | All collected data was collaboratively reviewed by the three research assistants to ensure accuracy, consistency and impartiality in the final evaluation. Key areas for app development and improvement were highlighted. Each assistant independently analyzed the data, a process which was followed by group discussions to resolve discrepancies and arrive at a consensus. This collaborative approach enhances the reliability of the findings by incorporating diverse perspectives and minimizing individual biases. | 6 |
| Data items | 11 | This included fundamental app details (such as its name, country of origin, developers, and user ratings), as well as more in-depth data such as app description, first launch time, pricing, overall comments, and the number of user comments. Additional data captured include the app’s stated aims, its main features, its target users, user experience and interaction logic, as well as functionalities related to the aforementioned cognitive support features of Capture Memory, Save, Reminisce, and Share. User interface and visual design elements (like color, imagery and screenshots) were also recorded and analyzed. | 6 |
| Critical appraisal of individual sources of evidence§ | 12 | Each team member independently documented information gained from extensive user testing of each app. | 6 |
| Synthesis of results | 13 | Framework analysis was then employed to systematically categorize specific features of digital storytelling apps (25), allowing for a structured comparison of more nuanced functionalities such as ‘Capture Memory’, ‘Save’, ‘Reminisce’, and ‘Share’, highlighting how these features support cognitive functions and user engagement. Statistical analysis was conducted based on the MARS (Mobile Application Rating Scale) (26) ratings to evaluate app quality. Mean scores were calculated across four domains: engagement, functionality, aesthetics, and information quality. This statistical approach provided a quantifiable measure of app utility and quality, facilitating objective comparisons. Thematic analysis was additionally utilized to gauge user feedback and engagement levels, a process which entailed an examination of user reviews to identify common sentiments, areas of satisfaction, and points of contention. This layer of analysis helps us to understand the user experience and the impact of app updates on user satisfaction. | 6 |
| **RESULTS** | | | |
| Selection of sources of evidence | 14 | Google Play yielded 178 apps, while iTunes had a slightly higher yield with 203 apps. After removing duplicates, the aggregate number of unique applications stood at 297. The screening process then ensued, during which 13 applications were found to be inaccessible; another 27 applications did not offer Chinese or English language options. | 7 |
| Characteristics of sources of evidence | 15 | An overview of the included storytelling and memory apps, detailing their platform compatibility, developer origins, user ratings, and feedback across both iOS and Android platforms. | 8 |
| Critical appraisal within sources of evidence | 16 | In order to provide a systematic quality assessment, our research evaluated a sample of the available storytelling applications using the Mobile Application Rating Scale (MARS) across four domains: Engagement, Functionality, Aesthetics, and Information, culminating in an overall score (see Table 5). | 13 |
| Results of individual sources of evidence | 17 | For each included source of evidence, present the relevant data that were charted Features of Digital Storytelling Apps, Quality Assessment of Digital Storytelling Apps: MARS Rating, and User Feedback and Engagement. | 7-15 |
| Synthesis of results | 18 | Summarize and/or present the charting results as they relate to the review questions and objectives. | 7-15 |
| **DISCUSSION** | | | |
| Summary of evidence | 19 | The principal findings from the evaluation of nine digital storytelling apps using the Mobile Application Rating Scale (MARS) offer significant insights into the app landscape. Among the apps evaluated, Memoirs of Life' and 'Memorize: Diaries, Memories, Notes, Ideas, Timelines, Categories' scored the highest, with ‘Grand Storyteller’ also performing well, indicating a balance in user engagement, functionality, aesthetics, and information quality. ‘Story Dice - Story Telling’ yielded the lowest mean score at 3.04, primarily due to its deficiencies in information quality. Apps with lower information quality scores are likely to struggle with user retention and credibility, which are critical for maintaining user interest. Users’ feedback on specific app features, and innovative features like the photo classification functionality received positive feedback, contrasting with criticisms of ‘Story Dice’ for its lack of innovation.  Apps such as ‘FamilySearch’ have received extensive positive feedback and high user engagement, whereas others like ‘Grand Storyteller’ have received minimal feedback, highlighting market penetration inconsistencies and varying user interest levels. The geographic distribution of app developers, with significant contributions from China and the USA, indicates a global interest in digital storytelling technologies.  In summary, while certain apps set benchmarks for excellence in the digital storytelling domain, others lag, particularly in crucial areas such as information quality. This disparity accentuates the need for ongoing improvements in app functionality and user interface to better cater to specific user groups, particularly those with cognitive impairments. | 15 |
| Limitations | 20 | Nonetheless, this study has a number of limitations. First, while the rating scale is effective in evaluating the current milieu of apps, it cannot replace the need for user-centered design and evidence-based practices in app development, especially within the health behavior sector. A significant limitation of this study is its geographical restriction to the Chinese market, meaning that apps that may be available in other regions have not been considered. This limitation may have resulted in the exclusion of potentially relevant apps not accessible within the Chinese market, thus limiting the scope of this review. Future research would do well to increase the geographical scope of analysis. Future studies should also focus on evaluating specific storytelling features and guided content within these apps, as there is currently no established standard for optimal storytelling practices, nor their efficacy in mitigating symptoms of cognitive impairment. | 19 |
| Conclusions | 21 | This systematic search and evaluation of digital storytelling apps in the Chinese app ecosystem reveals a gap in the quality and features of these applications, particularly for users with cognitive impairment. Our findings, derived from an extensive search in both English and Chinese on the Google Play Store and iTunes Store, identified 297 unique apps, with only 9 meeting our criteria for further evaluation. The apps sampled herein and evaluated using MARS, exhibited varied levels of quality. Key functionalities like capture memory, save, reminisce, and share was not uniformly present across all apps. The highest-rated apps were ‘Memoirs of Life’ and ‘Memorize: Diaries, Memories, Notes, Ideas, Timelines, Categories’, indicating a balance in engagement, functionality, and information quality. However, overall, many apps reveal significant room for improvement, especially in the information domain (where user complaints were concentrated). This exploratory study lays the foundation for future research and development in digital storytelling applications, aiming to enhance user experience and effectiveness. The significance of these results lies in guiding the development of more effective digital storytelling applications to maximize its therapeutic potential with a focus on the cognitively impaired. Concomitantly, the findings emphasize the need for improved content accuracy, user interface design, and the integration of AI technologies to enhance cognitive support and user engagement by aiding in photo restoration and material selection. | 20 |
| **FUNDING** | | | |
| Funding | 22 | Describe sources of funding for the included sources of evidence, as well as sources of funding for the scoping review. Describe the role of the funders of the scoping review. | Not applicable |

JBI = Joanna Briggs Institute; PRISMA-ScR = Preferred Reporting Items for Systematic reviews and Meta-Analyses extension for Scoping Reviews.

* Where *sources of evidence* (see second footnote) are compiled from, such as bibliographic databases, social media platforms, and Web sites.

† A more inclusive/heterogeneous term used to account for the different types of evidence or data sources (e.g., quantitative and/or qualitative research, expert opinion, and policy documents) that may be eligible in a scoping review as opposed to only studies. This is not to be confused with *information sources* (see first footnote).

‡ The frameworks by Arksey and O’Malley (6) and Levac and colleagues (7) and the JBI guidance (4, 5) refer to the process of data extraction in a scoping review as data charting*.*

§ The process of systematically examining research evidence to assess its validity, results, and relevance before using it to inform a decision. This term is used for items 12 and 19 instead of "risk of bias" (which is more applicable to systematic reviews of interventions) to include and acknowledge the various sources of evidence that may be used in a scoping review (e.g., quantitative and/or qualitative research, expert opinion, and policy document).

*From:* Tricco AC, Lillie E, Zarin W, O'Brien KK, Colquhoun H, Levac D, et al. PRISMA Extension for Scoping Reviews (PRISMAScR): Checklist and Explanation. Ann Intern Med. 2018;169:467–473. [doi: 10.7326/M18-0850](http://annals.org/aim/fullarticle/2700389/prisma-extension-scoping-reviews-prisma-scr-checklist-explanation).
